# Sustainable and Green Production of Nanostructured Cellulose by a 2-Step Mechano-Enzymatic Process

**DOI:** 10.3390/polym15051115

**Published:** 2023-02-23

**Authors:** Martina Aulitto, Rachele Castaldo, Roberto Avolio, Maria Emanuela Errico, Yong-Quan Xu, Gennaro Gentile, Patrizia Contursi

**Affiliations:** 1 Department of Biology, Università degli Studi di Napoli Federico II, 80138 Napoli, Italy; 2 Biological Systems and Engineering Division, Lawrence Berkeley National Laboratory, Berkeley, CA 94720, USA; 3 Institute of Polymers, Composites and Biomaterials (IPCB), National Research Council of Italy (CNR), 80078 Pozzuoli, Italy; 4 Tea Research Institute, Chinese Academy of Agricultural Sciences, 9P South Meiling Road, Hangzhou 310008, China

**Keywords:** nanostructured cellulose, enzymatic cocktails, ball milling, functional coating

## Abstract

Nanostructured cellulose (NC) represents an emerging sustainable biomaterial for diverse biotechnological applications; however, its production requires hazardous chemicals that render the process ecologically unfriendly. Using commercial plant-derived cellulose, an innovative strategy for NC production based on the combination of mechanical and enzymatic approaches was proposed as a sustainable alternative to conventional chemical procedures. After ball milling, the average length of the fibers was reduced by one order of magnitude (down to 10–20 μm) and the crystallinity index decreased from 0.54 to 0.07–0.18. Moreover, a 60 min ball milling pre-treatment followed by 3 h Cellic Ctec2 enzymatic hydrolysis led to NC production (15% yield). Analysis of the structural features of NC obtained by the mechano-enzymatic process revealed that the diameters of the obtained cellulose fibrils and particles were in the range of 200–500 nm and approximately 50 nm, respectively. Interestingly, the film-forming property on polyethylene (coating ≅ 2 μm thickness) was successfully demonstrated and a significant reduction (18%) of the oxygen transmission rate was obtained. Altogether, these findings demonstrated that nanostructured cellulose could be successfully produced using a novel, cheap, and rapid 2-step physico-enzymatic process that provides a potential green and sustainable route that could be exploitable in future biorefineries.

## 1. Introduction

Cellulose is one of the most abundant polymers on Earth and represents an intriguing building block for the production of functional materials from renewable non-fossil carbon sources [1,2]. Moreover, the cellulose structure is characterized by amorphous and crystalline portions that differ mainly in their chain length and hydrogen bonding pattern [3] and the relative amounts of which depend on the isolation source. Crystalline cellulose exhibits long, well-packed chains via strong hydrogen bonds and Van der Waals interactions; instead, amorphous cellulose can vary in length from long to very short chains that twist and bend, thus altering the ordered arrangement [4]. Since the physical properties of cellulose, as well as its chemical behavior and reactivity, are affected by its ultrastructure, ad hoc mechanical and chemical treatments are currently used to exploit its biotechnological applications [5]. Specifically, cellulose-based materials, available at low cost when isolated from waste biomass, have gained attention for the production of nanostructured cellulose (NC) [6]. NC can be categorized into nanostructured materials (microcrystalline cellulose and cellulose microfibrils) and nanofibers, such as bacterial cellulose, cellulose nanocrystals (CNC), and cellulose nanofibrils (CNF) [7,8,9]. In particular, CNC are usually rod-like nanoparticles 4–70 nm in width, 100–6000 nm in length, with a crystallinity index of 0.54–0.88. In contrast, CNF are longer and wider nanofibers (20–100 nm in width and >10,000 nm in length), with a lower crystallinity content [10,11]. CNC are produced from diverse biological sources mainly by strong acid hydrolysis that removes amorphous cellulose from the source material, thus producing high purity cellulose crystals [7]. CNF, on the other hand, are produced by TEMPO-mediated oxidation (2,2,6,6,-tetramethylpipelidine-1-oxyl radical), high-pressure homogenization, mechanical fibrillation, or enzymatic hydrolysis. The morphology and dimensions of CNF can vary significantly, depending on the treatment used [12].

Similar to native cellulose, NC exhibits desirable properties such as high strength, stiffness, and biodegradability. The peculiar features of NC have been exploited in electronics [13], as reinforcement agents in polymer composite materials [14], as well as in water treatment for the removal of heavy metal ions and organic pollutants [1,15,16]. Moreover, due to their biocompatibility and non-toxicity, cellulose films and membranes are exploited as wound dressing agents [17] and in the packaging field to meet the increasing need for biodegradable and renewable materials [18]. To use NC in engineering applications, specific features and functions, such as uniformity in size, are required. Currently, methods to achieve nanometer-sized cellulose are based either on chemical treatments with organic acids, ionic liquids, and supercritical water, or physical processing such as micro-grinding, high-pressure microfluidization, and cavitation using ultrasound [19]. These approaches have been applied to extract NC from diverse biomasses, such as filter papers, cotton linters, Eucalyptus hardwood, broomcorn stalks, nata de coco, palm oil, wood sawdust, etc. [7].

At the industrial scale, NC is produced by Nippon^®^ and Celluforce^®^ through oxidation mediated by 2,2C,6,6- tetramethylpiperidine-1-oxyl radical (TEMPO), followed by mechanical defibrillation [20,21]. Although oxidation reactions are largely employed, the use of TEMPO reagent together with high concentrations of salts and hypochlorite does not make this process environmentally sustainable or economically feasible since expensive materials resistant to corrosive agent (e.g., titanium) are necessary [6]. Therefore, the research of new green technologies for NC extraction, such as enzyme-based strategies employing cellulase activities, are strongly required. Specifically, enzymatic hydrolysis of cellulose is a complex mechanism involving the synergistic action of several mesophilic and/or thermophilic enzymes to break glycoside-type bonds, i.e., exoglucanases (CHBs), β-glucosidases (BGs), endoglucanases (EGs), and lytic polysaccharide monooxygenases (LPMOs) [22]. EGs exert their hydrolytic activity on the internal bonds of cellulose, while CHBs act on its crystalline ends, releasing cellobiose that is broken down into molecules of glucose by BGs [23]. Interestingly, LPMOs, which are more recently discovered, participate in cellulose degradation through oxidative reactions [24]. So far, the use of cellulases for the production of NC has not been well established since crystalline domains affect hydrolysis efficiency and kinetics due to their lower accessibility to water molecules [25]. In this context, previous studies have proven that ball milling represents an easy process to decrease the crystallinity degree of cellulose [26]. This approach has been already applied to obtain polymer/cellulose composites showing improved biodegradability [27,28,29].

The objective of this work was to develop a green process based on the combination of ball milling amorphization of cellulose fibers with the hydrolytic action of thermophilic enzyme cocktails for the sustainable and easily scalable production of NC. Notably, these cellulose nanostructures exhibited tailored morphology, size distribution, and surface properties suitable for gas barrier applications.

## 2. Methods

### 2.1. Reagents

Cellulose Arbocel^®^ was kindly supplied by JRS (Rosenberg, Germany). Two samples of cellulose were employed, with average fibers length of 700 and 200 µm. These samples are indicated in the text as C700 and C200, respectively. Carboxymethyl cellulose (CMC) was purchased from Sigma (St. Louis, MO, USA). The enzymes used in this work were either single enzymes (all from Megazyme, Wicklow, Ireland) i.e., endo-1-4-beta-D-glucanase (*Thermotoga maritima*, Megazyme), endo-1-4-beta-D-glucanase (*Bacillus amyloliquefaciens*, Megazyme,), endo-1,4-β-D-glucanase (*Thermobifida halotolerans*, Megazyme) or enzymatic cocktails (all from Sigma, St. Louise, Missouri, USA), i.e., cellulases from *Trichoderma reesei* ATCC 26921, Viscozyme^®^ L, and Cellic Ctec2. Polyethylene flexible packaging films (PE) were supplied by Manuli Films (Sessa Aurunca, Italy).

### 2.2. Ball Milling

Cellulose sample C200 was milled using a Retsch PM100 planetary ball milling system (Haan, Germany) with a 125 mL steel milling cup and 25 spheres, each 10 mm in diameter. Before milling, the cellulose was dried at 90 °C under vacuum for 24 h. The sphere:cellulose weight ratio was set at 10:1. The ball milling process was carried out for 30 and 60 min, obtaining the samples indicated as BM30 and BM60, respectively.

### 2.3. Enzymatic Treatments and Carbohydrate Detection Assay

To set optimal conditions for enzymatic cellulose digestion, different ratios of cellulose (C700, C200, BM30, BM60) and enzyme(s) were tested. Specifically, the amount of single enzyme or enzymatic cocktail ranged from 5 to 100 hydrolytic units, whereas the cellulose amount varied from 5 to 50 mg. The reactions were conducted in 0.1 M citrate buffer, pH 5, or in water, in static or dynamic conditions (orbital shaking 180 rpm), at 37 or 50 °C, in a time frame from 0.5 h to overnight incubation (16 h). Upon enzymatic digestion, glucose release was monitored through the 3,5-dinitrosalicylic acid (DNS) assay, which detects the presence of free carbonyl groups (C=O) of reducing sugars through oxidation of the aldehyde functional group. The reaction was performed in polystyrene 96-well plates. Increasing amounts of glucose to be used as standards contained 0, 0.2, 0.4, 0.6, 0.8, and 1.0 µmole of D (+)-glucose monohydrate (Sigma) in 40 µL of distilled water. Then, 160 µL of DNS solution was added into each well, yielding a final volume of 200 µL, and incubated for 30 min at 100 °C. The glucose released after enzyme digestion was evaluated by substituting the standard solution with an identical volume of the enzymatic reaction. Finally, the test plates were analyzed using a microplate reader (Synergy H4; Bio-TEK Instruments, Winooski, VT, USA) to determine the optical density of each sample at 570 nm. The efficiency of degradation was also evaluated through measurement of the dry weight of cellulose after enzymatic digestion. Samples were centrifuged for 15 min at 6000 rpm at 4 °C and the pellet was washed in MilliQ water several times to get rid of the residual cellulose from the components present in the enzymatic reactions. The absence of enzyme(s) in the preparation was monitored through spectrophotometric analysis at 280 and 220 nm. The pellet was dried at 50 °C overnight and the dry weight was calculated.

### 2.4. Characterization of NC

The crystallinity of the pristine and ball-milled cellulose samples was investigated by solid state NMR acquiring ^13^C spectra in cross polarization-magic angle spinning (CPMAS) mode. Spectra were collected at 100.47 MHz on a Bruker Avance II 400 (Bruker Biospin, Billerica, MA, USA) spectrometer operating at a static field of 9.4 T, equipped with a 4 mm MAS probe. Neat and ball-milled cellulose samples were packed into 4 mm zirconia rotors and sealed with Kel-F caps. The spinning speed was set at 8 kHz for all NMR experiments. Spectra were acquired with a contact time of 1.5 ms, a 1 H π/2 pulse width of 3.0 µs, collecting 10.000 scans. Spectral deconvolution was performed using the Grams/8.0AI software package (THERMO Electron Corporation, Waltham, MA, USA). A mixed Gaussian-Lorentzian (Lorentzian ≤15%) line shape was chosen. Each resonance was characterized by the following parameters: amplitude, chemical shift, and line width at half height. The morphology of the pristine and digested cellulose samples was investigated by scanning electron microscopy (SEM) using a FEI Quanta 200 FEG SEM (FEI, Eindhoven, The Netherlands). Before the SEM observations, the cellulose samples were sputter coated with a 10 nm-thick Au-Pd layer. All samples were observed at 10 kV acceleration voltage using a secondary electron detector. The BM60-3h sample was also analyzed by bright-field transmission electron microscopy (TEM) using a FEI Tecnai G12 Spirit Twin TEM (LaB6 source) at 120 kV acceleration voltage (FEI, Eindhoven, The Netherlands), equipped with a FEI Eagle 4 k CCD camera. The sample was collected on carbon coated copper grids, which were submerged into an aqueous suspension of BM60-3h. SEM and TEM images were analyzed using ImageJ (Public Domain, BSD-2) software to measure the diameters of the cellulose fibrils and particles. At least 40 fibrils and nanoparticles of the BM60-3h sample were measured to assess their average diameter distribution.

BM60-3h was analyzed by Fourier transform infrared (FTIR) spectroscopy in attenuated total reflectance (ATR) mode, using a PerkinElmer Spectrum One (Waltham, MA, USA) FTIR spectrometer equipped with an ATR module, with a resolution of 4 cm^−1^ collecting 32 scan, and by thermogravimetric analysis (TGA). The latter was performed by heating the sample from 100 to 800 °C at a rate of 10 °C/min under a nitrogen atmosphere, using a PerkinElmer Pyris 1 TG/DTA (Waltham, MA, USA).

### 2.5. Application of NC in Functional Coating

Digested sample BM60-3h was employed to obtain a thin film on a PE substrate by rod coating. Before application of the coating, BM60-3h was dispersed in distilled water using a Transsonic Digitals ultrasonic bath for 15 min. The PE-BM60-3h sample was characterized by SEM analysis. The UV-visible spectra of PE and PE-BM60-3h were collected using a V-570 UV spectrophotometer (Jasco, Easton, PA, USA) in the 200–800 nm wavelength range and with 0.5 nm resolution. The oxygen permeability of coated PE in comparison to that of the pristine film was characterized through analysis of the oxygen transmission rate at 25 °C and 50% relative humidity (RH), using a Multiperm Extrasolution Permeabilimeter (PermTech, Pieve Fosciana, Italy). Triplicate measurements of UV-Vis and oxygen permeability were performed in different areas of the samples.

## 3. Results and Discussion

### 3.1. Morphological Analysis and Enzymatic Treatment of Pristine Cellulose

**Figure 1:**
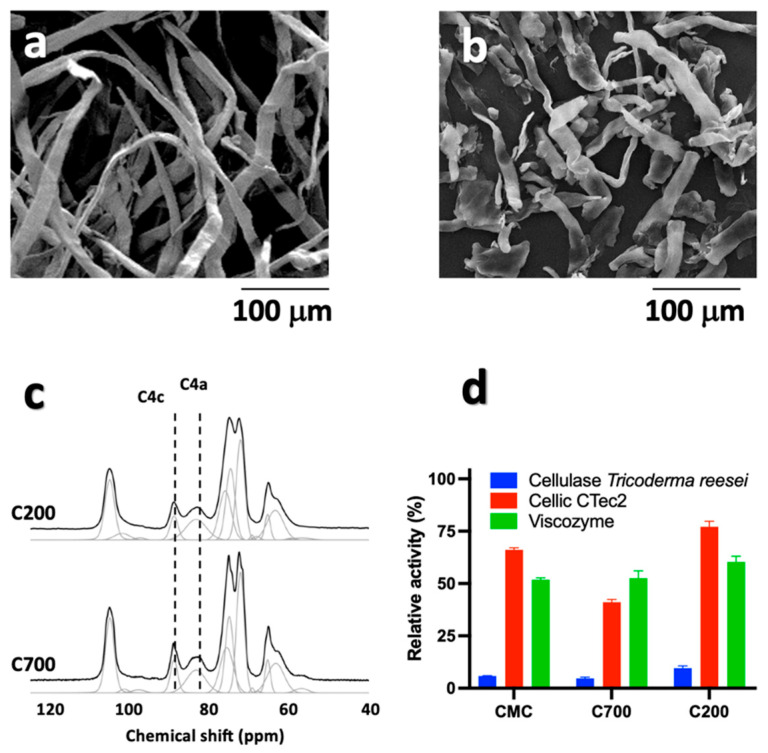
SEM images of (**a**) C700 and (**b**) C200. In panel (**c**), ^13^C CP-MAS NMR spectra of C200 and C700 samples are shown. The resonances used for quantitative evaluation of the crystallinity index (C4c and C4a) are highlighted. (**d**) Relative activity of treatment of cellulase from *T. reesei*, Viscozyme^®^ L, and Cellic Ctec2 on CMC, C200, and C700.

Two commercial crystalline cellulose samples (C700 and C200) were characterized at the morphological level. Specifically, SEM analysis (Figure 1a,b) revealed similar diameters (approximately 15 µm) and different lengths of the fibers (approximately 700 and 200 μm), with length/diameter ratios of approximately 46.6 and 13.3 for C700 and C200, respectively.

The crystallinity of C700 and C200 was assessed through ^13^C NMR analysis. In particular, the peaks referring to C4 atoms centered at 88.9 ppm (C4c for crystalline domains) and 83 ppm (C4a for amorphous domains) were selected as references for the quantitative evaluation. Comparing the area of these deconvoluted resonances, crystallinity index values of 0.59 and 0.54 were determined for C700 and C200, respectively.

**Figure 2:**
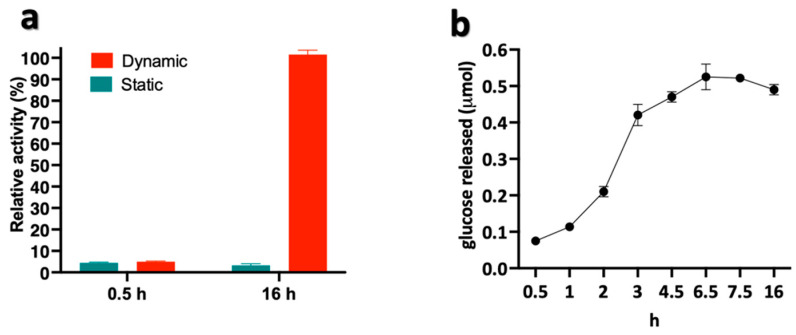
C200 sample treated with Cellic Ctec2. (**a**) Enzymatic hydrolysis under dynamic and static conditions; (**b**) Kinetic analysis of degradation process under dynamic conditions.

These two samples were hydrolyzed using thermophilic enzymes to investigate the substrate accessibility and formation of NC under controlled conditions. As a first trial, we selected commercial thermophilic endoglucanases, which are mainly responsible for the degradation of the amorphous fraction of the cellulose [30]. In general, extremophilic microorganisms and their viruses thrive under harsh chemical-physical conditions (high temperature, low pH, etc.) and exhibit an efficient degradative metabolism towards complex carbohydrates such as those contained in cellulose/hemicellulose biomasses [31,32,33]. Moreover, thermophilic enzymes work better on complex matrices since the high temperature increases substrate accessibility and enzymatic attack [34]. A number of hydrolytic enzymes from hot environments have been identified through genomic and metagenomic analysis and most of the commercially available cellulase enzymes are thermophilic or moderately thermophilic [35,36,37]. Three different endo-1-4-beta-D-glucanases (from *Thermotoga maritima*, *Bacillus amyloliquefaciens*, and *Thermobifida halotolerans*) with optimal temperatures ranging from 40 to 80 °C were used on C700 and C200. Different incubation times (from 1 to 24 h) and various enzyme:substrate ratios were tested (data not shown). The downstream analysis performed through DNS assays revealed that no reducing sugars were released under these conditions. This result was traced back to the ultrastructural difference between the micrometric C700 and C200 cellulose compared to the reference substrate (CMC) taken as a positive control for the hydrolytic activity. To overcome this issue, enzymatic mixtures were used in place of single enzymes, i.e., cellulases from *Trichoderma reesei*, Viscozyme^®^ L, and Cellic Ctec2. Cellulase cocktails from *T. reesei* are produced by submerged fermentation and are routinely utilized to investigate the biodegradability of bioabsorbable bacterial cellulose. The mixture is mainly composed of two exoglucanases, at least five endoglucanases, and cellobiases [38]. In contrast, Viscozyme^®^ L is obtained from fungal *Aspergillus* spp. and contains several hemicellulases along with cellulase activities [38]. A similar composition is reported for Cellic Ctec2, an enzymatic blend extensively employed in the conversion of pre-treated lignocellulosic biomass materials into fermentable sugars for application in biofuel and biochemical research. The three enzymatic cocktails were tested in comparison on C200, C700, and commercial CMC using a 10 mL mixture containing 1.5 mg/mL of cellulose supplemented with 0.4 filter paper unit of each enzymatic blend, calculated according to the NREL method [39]. As shown in Figure 1d, the degradation activity of Cellic CTec2 was generally higher than that of Viscozyme and utterly more efficient than that of the cellulase from *T. reesei* (Figure 1). Therefore, all the subsequent analyses were carried out with Cellic CTec2 on C200 (Figure 2). This sample was selected because of its major accessibility to the enzymatic treatment compared to C700.

In order to set up tunable conditions for the enzymatic digestion, we compared static vs. dynamic incubation. The results shown in Figure 2a highlight that the amount of glucose was much higher under stirring conditions and increased up to 20-fold after 16 h of incubation. Conversely, we did not observe any relevant difference when the sample was incubated under static or dynamic conditions for a short time window. This result indicated that the dispersion of the insoluble cellulose substrate achieved under continuous stirring along with prolonged exposure to enzymatic attack increased the process efficiency. Furthermore, we compared two different temperatures, i.e., 37 vs. 50 °C, the latter being the optimal temperature of Cellic Ctec2. After 16 h of incubation (end point of the assay), the amount of sugars released did not change, suggesting that the digestion was complete after 16 h regardless of the temperature used (data not shown). Interestingly, the digestion efficiency increased progressively, peaking at 6.5 h and slightly decreasing afterwards, possibly as a consequence of product inhibition and/or limits in the detection assay (Figure 2b). Overall, these results showed that the action of the Cellic Ctec2 cocktail on cellulose was tunable by varying the exposure time and stirring conditions during digestion.

### 3.2. Ball Milling Treatment of Cellulose Samples

**Figure 3:**
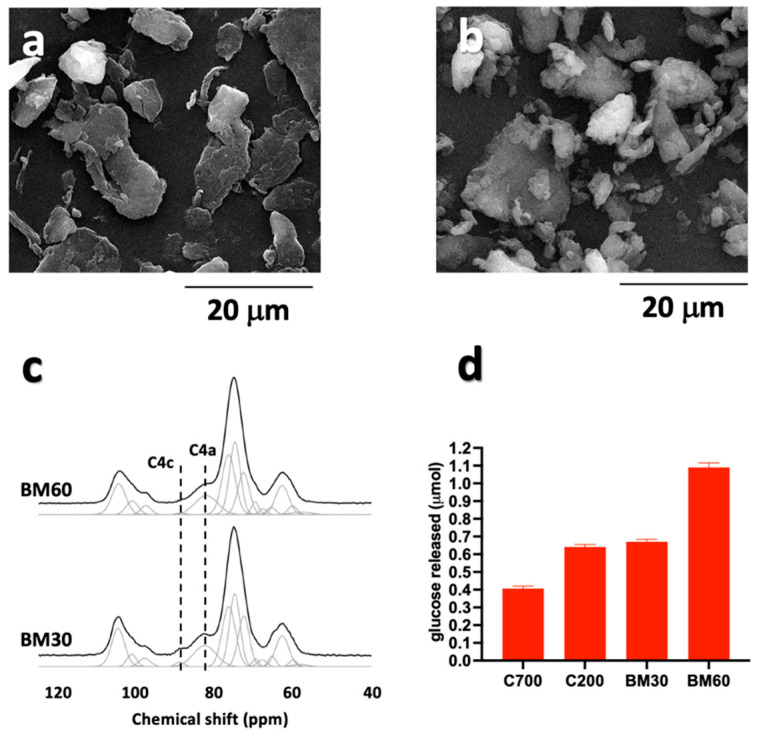
SEM images of (**a**) BM30 and (**b**) BM60. In panel (**c**), the ^13^C CP-MAS NMR spectra of BM30 and BM60. The resonances used for quantitative evaluation of the crystallinity index (C4c and C4a) are reported. (**d**) Enzymatic hydrolysis of C700, C200, BM30, and BM60 celluloses mediated by Cellic Ctec2 under optimal conditions.

To test the feasibility of the ball milling process to improve the accessibility of cellulose to the enzymatic treatment, the C200 sample was chosen because of its reduced fiber length, low crystallinity degree, as well as its sensitivity to enzymatic hydrolysis (Figure 1 and Figure 2). The 30 min (BM30) and 60 min (BM60) treatment led to a morphological transition from a fibrous-like to particle-like structure, with average lateral dimensions ranging between 10 and 20 μm. This result pointed to a significant reduction of the particles size compared to the pristine fibers of the C200 sample. As a consequence, the ratio between the maximum and minimum lateral size of the NC turned out to be 1.8 for BM30 and 1.5 for BM60. Moreover, a reduction of the crystallinity index from 0.54 (untreated cellulose) to 0.18 and 0.07 for BM30 and BM60, respectively, was assessed through solid-state NMR (Figure 3c).

To compare the efficiency of the enzymatic digestion on pristine and ball-milled samples, C700, C200, BM30, and BM60 were treated with Cellic Ctec2 under the optimal conditions set above (37 °C under shaking ON). As expected (Figure 3d), the amount of glucose released was inversely related to the crystallinity index of the samples, which was lowest for the BM60 sample and highest for the C700 sample, thus confirming the efficiency of the ball milling pre-treatment to enhance the subsequent enzymatic process.

### 3.3. Optimization of the 2-Step Mechano-Enzymatic Process

**Figure 4:**
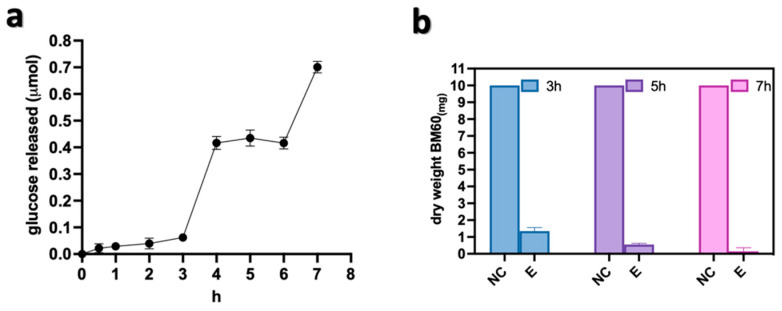
Time course of the enzymatic digestion on BM60 sample. (**a**) Kinetic curve of released glucose and (**b**) dry weight measurements of the residual cellulose. NC: negative control (without Cellic Ctec2) and E: with Cellic Ctec2.

Once established that the ball milling treatment increased the efficiency of the enzymatic digestion, a time course of hydrolysis was performed to optimize the mechano-enzymatic process on the BM60 sample. The kinetic analysis showed that the highest glucose amount was released after 7 h of incubation (Figure 4a), when the complete digestion of the cellulose was achieved as inferred by the negligible percentage of its residual dry weight (Figure 4b).

**Figure 5:**
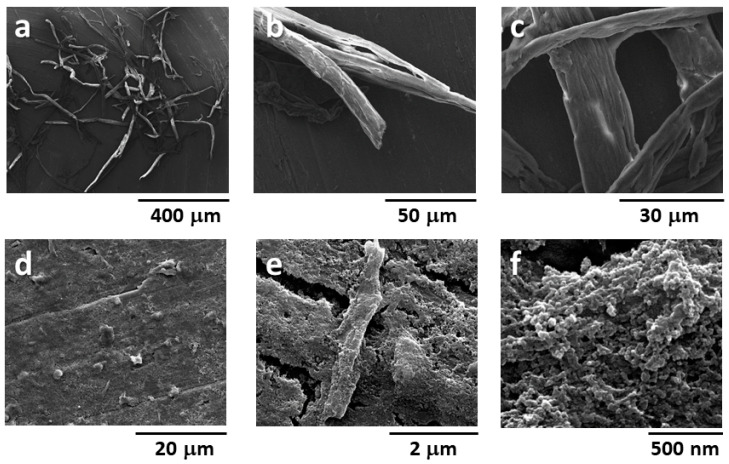
SEM images of C700-3h (**a**–**c**), BM60-3h (**d**–**f**).

Then, a scale-up of the reaction mixture from 10 to 150 mg of BM60 cellulose was performed after 3 h of incubation in order to avoid the complete digestion of cellulose. Interestingly, a 15% yield was achieved and the so-called BM60-3h sample was used for subsequent characterization. Morphologic analysis was performed by SEM on C700-3h and BM60-3h because of their significant differences in terms of morphology and crystallinity degree. As shown in Figure 5a–c, the fiber morphology of C700 was only partially affected by the enzymatic treatment, since the average diameter and length of the fibers were unchanged after digestion. In contrast, the morphology of BM60-3h was strongly modified by the enzymatic treatment (Figure 5d–f). Indeed, it showed a highly defibrillated morphology characterized by the presence of fibers with submicrometer diameters, lengths between 500 nm and 3 µm (cellulose fibrils), and quasi-spherical particles with a diameter lower than 100 nm (cellulose nanoparticles).

**Figure 6:**
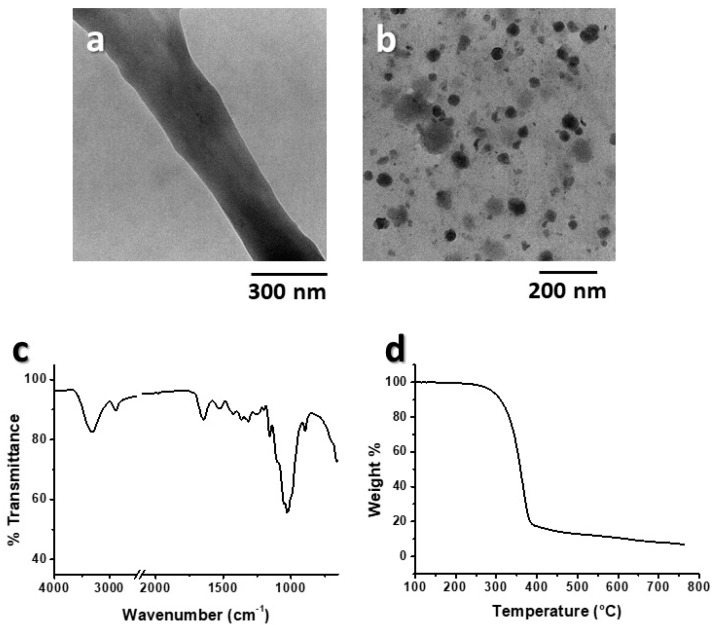
Bright field TEM images (**a**,**b**) FTIR spectrum (**c**) and TGA trace (**d**) of BM60-3h.

A more in-depth analysis of BM60-3h was performed by TEM to enable precise measurements of fibril diameter (200–500 nm, Figure 6a) and nanoparticles (50 nm, Figure 6b). FTIR analysis (Figure 6c) showed the characteristic signals of cellulose: (i) the absorption band centered at 3330 cm^−1^ was assigned to hydroxyl group stretching; (ii) the bands at 2906 cm^−1^ and 1373 cm^−1^ were associated with stretching and deformation vibrations of C-H groups in glucose units; (iii) the absorption band at 1654 C=O was associated with stretching vibrations; (iv) the band at 1526 cm^−1^ was associated with aromatic skeletal vibrations; and (v) the absorption band at 1050 cm^−1^ was associated with -C-O- group vibrations [40].

TGA analysis of BM60-3h (Figure 6d) showed a significant thermal degradation event at approximately 350 °C, which was associated with the carbonization process of cellulose, with a weight loss curve similar to that of high purity cellulose [41], indicating that the main chemical features of cellulose and NC were unaltered.

Taken together, these results pointed to the successful combination of ball milling with tunable enzymatic treatments to achieve suitable amounts of highly pure NC.

### 3.4. Film-Forming Property of Mechano-Enzymatic Treated Cellulose

**Figure 7:**
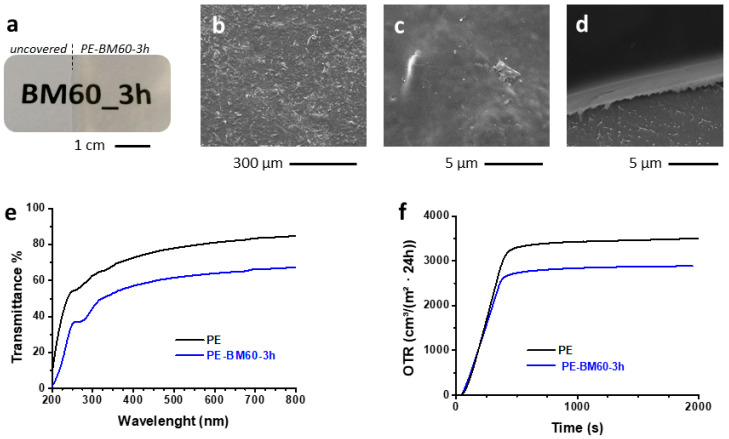
Picture of the overlayed PE-BM60-3h and PE samples on text printed on white paper (**a**) and SEM images (**b**–**d**) of PE-BM60-3h. UV spectra (**e**) and OTR measurements at 25 °C and 50% RH (**f**) of PE and PE-BM60-3h.

Food packaging biomaterials are attractive alternatives to conventional non degradable petrochemical-based polymers [19]. The use of NC for food packaging is desirable because of its ability to form a dense percolating network due to hydrogen bonds with low gas permeability [18]. The peculiar nanofibrous/nanoparticle morphology of BM60-3h dispersed in water was investigated to test its film-forming property in coating applications. A thin coating, with a nominal thickness of approximately 2 μm and showing excellent film-forming capability, was obtained on PE by rod coating and NC water dispersion. Interestingly, BM60-3h self-assembled in a regular, homogeneous, and transparent film when water casting was performed at room temperature, as shown in Figure 7a. The transparency of BM60-3h was evaluated by comparing the transmittance (T%) of PE-BM60-3h and PE samples within the UV-Vis wavelength range (200–800 nm). Notably, the moderate reduction of T% values from PE (>72%) to PE-BM60-3h (>57%) in the visible range (400–800 nm) indicated that the transmittance of the PE substrate treated with the NC cellulose coating remained consistent for applicative purposes (Figure 7e). Such features were comparable to coatings obtained by TEMPO-mediated oxidation with smaller diameter cellulose nanofibers (3–4 nm in width) derived from softwood and hardwood celluloses. Moreover, these coatings exhibited a larger thickness (20 µm) and transmittance values of 90% (softwood) or 78% (hardwood) at 600 nm [42]. Interestingly, the BM60-3h coating with a thickness of approximately 2 µm obtained in this work through the proposed mechano-enzymatic process was characterized by transmittance of approximately 79% with respect to the uncoated substrate in the whole visible range.

The self-assembling property of NC is related to the formation of stable hydrogen bonds between the nanostructures as a consequence of water evaporation [43]. Morphological analysis of the coating revealed that the surface was characterized only by small diffuse wrinkles due to overlapping of cellulose fibrils (Figure 6b), thus indicating that the roughness of the film was overall negligible (Figure 6c). Moreover, the nominal thickness of the coating was confirmed through measurement of the section by SEM analysis, which showed, in particular, a thickness of 1.82 ± 0.06 µm in the area observed in Figure 6d.

In general, the applications of NC as water and gas barrier coatings are limited by its hydrophilicity. However, cellulose-based coatings exhibited interesting gas barrier properties when cellulose was nanosized [18,44]. For example, an OTR reduction of approximately 60% was obtained by applying a nanocellulose coating approximately 4 µm thick on biaxially oriented polypropylene/low density polyethylene laminates [45]. In our study, oxygen permeability tests were performed on PE and PE-BM60-3h to evaluate the potential use of BM60-3h for functional coating applications. A 2 µm-thick coating led to an interesting reduction of the oxygen transmission rate of the sample (18%) compared to pristine PE (Figure 6d). These results supported the proof-of-concept that NC obtained through the 2-step mechano-enzymatic process described herein is suitable for gas barrier applications. A further improvement of these properties could be achieved by chemically modifying the cellulose to increase the hydrophobicity of the NC material. Alternatively, a layered system (i.e., sandwiched or so-called multilayer packaging films) can be envisaged to protect NC from the effects of moisture [46].

## 4. Conclusions

The commonly used methods for NC extraction from cellulose materials generate a high amount of acidic wastewater from acid hydrolysis, lead to high energy consumption, and have long reaction times. It is known that ball milling is an effective mechanical method for the extraction of NC from biomass, while enzymatic hydrolysis of cellulose is specific and generally requires mild conditions and limited energy consumption. A number of studies addressing NC extraction from diverse biomasses using one of these two methods are described in literature [7,14]. However, comprehensive studies on how these two physico-biochemical approaches can be tuned and intersected to synergistically optimize NC production in the context of the circular economy (i.e., from renewable biomasses) are missing. This study demonstrates the feasibility of the two-step mechano-enzymatic approach for the production of NC under rapid, cheap, and environmentally sustainable conditions. Specifically, ball milling treatment caused significant morphological and ultrastructural modifications of the sample, leading to the formation of micrometer-sized cellulose particles that were further defibrillated upon enzymatic digestion. Interestingly, the method can be translated to extract cellulose from complex agri-food and lignocellulose feedstocks since the enzymatic cocktail used in this study contained an arsenal of hydrolytic enzymes effective on all constituents of these biomasses. Furthermore, the sugars released during NC production can be utilized as a carbon source during microbial fermentation by the biofuel and bioproduct industries. Notably, the NC obtained using this method was suitable as a film-forming biomaterial and can be employed as a packaging film combined with other polymers. Considering the wide spectrum of applications of NC in bioplastics, biomedicals, optics, and adhesives, this work represents a proof-of-concept for the easy, cheap, and sustainable production of a biotechnologically relevant nanomaterial.

## Data Availability

Not applicable.
